# Open label pilot of personalized, neuroimaging-guided theta burst stimulation in early-stage Alzheimer’s disease

**DOI:** 10.3389/fnins.2024.1492428

**Published:** 2024-12-09

**Authors:** Bhavani Kashyap, Leah R. Hanson, Sally K. Gustafson, Terry Barclay, Clarissa M. Howe, Samantha J. Sherman, Marcel Hungs, Michael H. Rosenbloom

**Affiliations:** ^1^HealthPartners Institute, Bloomington, MN, United States; ^2^HealthPartners Center for Memory and Aging, St Paul, MN, United States; ^3^Memory and Brain Wellness Center, University of Washington, Seattle, WA, United States; ^4^Department of Neurology, University of Washington, Seattle, WA, United States; ^5^University of Washington Alzheimer’s Disease Research Center, Seattle, WA, United States

**Keywords:** transcranial magnetic stimulation, clinical trial, functional connectivity, large-scale brain networks, human connectome project

## Abstract

**Background:**

Alzheimer’s disease (AD) is characterized by cerebral amyloid plaques and neurofibrillary tangles and disruption of large-scale brain networks (LSBNs). Transcranial magnetic stimulation (TMS) has emerged as a potential non-invasive AD treatment that may serve as an adjunct therapy with FDA approved medications.

**Methods:**

We conducted a 10-subject open label, single site study evaluating the effect of functional connectivity-resting state functional MRI guided-approach to TMS targeting with dysfunctional LSBNs in subjects with biomarker-confirmed early-stage AD (https://clinicaltrials.gov/study/NCT05292222). Subjects underwent pre-post imaging and testing to assess connectivity dysfunction and cognition. All participants received intermittent theta burst stimulation [(iTBS), (80% motor threshold; 5 sessions per day; 5 days; 3 targets; 18,000 pulses/day)] over 2 weeks. Three Human Connectome Project (HCP) defined parcellations were targeted, with one common right temporal area G dorsal (RTGd) target across all subjects and two personalized.

**Results:**

We identified the following parcellations to be dysfunctional: RTGd, left area 8A ventral (L8Av), left area 8B lateral (L8BL), and left area 55b (L55b). There were no changes in these parcellations after treatment, but subjects showed improvement on the Repeatable Battery for the Assessment of Neuropsychological Status attention index (9.7; *p* = 0.01). No subject dropped out of the treatment, though 3 participants were unable to tolerate the RTGd target due to facial twitching (*n* = 2) and anxiety (*n* = 1).

**Conclusion:**

Accelerated iTBS protocol was well-tolerated and personalized target-based treatment is feasible in early-stage AD. Further sham-controlled clinical trials are necessary to determine if this is an effective adjunctive treatment in early-stage AD.

## Introduction

Alzheimer’s disease (AD) is a progressive neurodegenerative condition characterized by the development of cerebral amyloid plaques, neurofibrillary tangles, and disruption of largescale brain networks (LSBNs) that affects nearly seven million Americans (2024) Alzheimer’s Disease Facts and Figures (2024). Until recently, the mainstay treatments for this condition have included cholinesterase inhibitors and memantine, neither of which have been found to impact the progression of Alzheimer’s disease. The development of anti-amyloid monoclonal antibodies (AMAs) has resulted in novel treatments that remove cerebral amyloid while reducing progression in cognitive and functional decline (Dyck et al., 2023; Sims et al., 2023). While these treatments represent a significant milestone in AD management, they are limited by infusion related reactions in up to 26% of patients (Dyck et al., 2023) and amyloid-related imaging abnormalities ranging from 21.5–40% (Dyck et al., 2023; Salloway et al., 2022). Furthermore, these drugs are primarily designed to impact disease progression (Cummings, 2023) rather than directly restore dysfunctional large scale brain networks (LSBNs).

An-FDA cleared treatment for refractory depression (Janicak and Dokucu, 2015), repetitive transcranial magnetic stimulation (rTMS) has significant potential as a non-invasive therapeutic for Alzheimer’s disease and related disorder (ADRD) and presents minimal risk in terms of adverse side effects. Currently, TMS is also FDA-cleared for the treatment of migraine with aura (Dodick et al., 2010), obsessive compulsive disorder (Carmi et al., 2019), and nicotine use disorder (Zangen et al., 2021) and is being explored as treatment for other neurological disorders such as stroke (Zhu et al., 2024), and Parkinson’s disease (Yang et al., 2024). A proposed mechanism of TMS action is that persistent enhancement of cortical excitability results in long-term potentiation (Huang et al., 2005) and increased expression of brain derived neurotrophic factors (Phillips et al., 1991). Repetitive TMS at 10 or 20 Hz applied to the dorsolateral prefrontal cortex (DLPFC), which is typically targeted for depression has resulted in cognitive benefits, such as everyday memory, or global cognition (Marra et al., 2015; Li et al., 2024). Furthermore, clinical trials have been performed in mild–moderate AD, demonstrating that intensive and maintenance to the precuneus with rTMS slows cognitive and functional decline as measured by the Clinical Dementia Rating- Sum of Boxes (CDR-SB) over a 6-month period (Koch et al., 2022) or with iTBS to the left dorsolateral prefrontal cortex (DLPFC) over 1 year as measured by the MOCA (Wu et al., 2024).

One potential therapeutic approach to ADRD is to leverage the role of functional connectivity using resting state functional magnetic resonance imaging (fc-rs-fMRI) with TMS to provide a precision-medicine approach to dysfunctional LSBNs. Through prior fc-rs-fMRI studies, we determined that temporal area G dorsal (TGd), a region defined by the Human Connectome Project (Baker et al., 2018; Glasser et al., 2016), frequently showed functional connectivity anomalies (high functional anomaly burden) in both AD and DLB (Rosenbloom et al., 2021). In addition, this region has been shown to be implicated in language processing and semantic dementia (Briggs et al., 2018b). Thus, our prior work identified this region as a particular TMS target. To allow for a precision-medicine approach to each patient, we also were interested in applying stimulation to other anomalous parcellations identified with fc-rs-fMRI.

We performed a pilot study evaluating the effect of combining an fc-rs-fMRI guided approach with intermittent theta burst (iTBS) TMS in early-stage AD subjects. Our primary aim was to estimate the effect of fc-rs-fMRI -guided iTBS TMS on connectivity dysfunction within the right temporal area G dorsal (RTGd) in persons with early-stage AD. We were also interested in evaluating the effect of fc-rs-fMRI -guided TBS on two additional dysfunctional parcellations within the default modal network (DMN), central executive network (CEN) and salience network (SN). Finally, we evaluated whether this approach impacted cognitive performance on the Repeatable Battery for the Assessment of Neuropsychological Status (RBANS) and the Geriatric Depression Score (GDS) in persons with early-stage AD.

## Methods

### Study design

**Figure 1:**
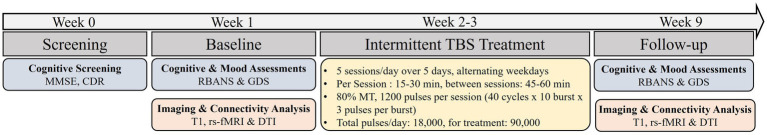
Study design and visits. Figure provides details regarding participants measures and workflow for the study. MMSE, mini mental status examination; CDR, clinical dementia rating; RBANS, repeatable battery for the assessment of neuropsychological status, GDS, Geriatric Depression Scale; T1, structural imaging; rs-fMRI, resting state functional magnetic resonance imaging; DTI, diffusion tensor imaging; TBS, theta burst stimulation; MT, motor threshold.

We conducted an open label, single arm pilot clinical trial to target LSBNs in early-stage AD using personalized TMS with neuronavigation. Participants received multi-modal imaging, and cognitive testing at baseline and at 6-weeks after treatment (see Figure 1). All participants were treated with an accelerated protocol of intermittent theta burst stimulation (iTBS). Additionally, participants were monitored for any adverse or serious adverse events during treatment including for seizure and syncope. The study was conducted within HealthPartners Center for Memory & Aging and approved by the local Institutional Review Board (A21-251) and registered with clinicaltrials.gov (NCT05292222).

### Study participants

Study participants were recruited over 17 months from a single specialty dementia care clinic in the upper Midwest. All participants were aged between 40 and 90 years with an established diagnosis of Mild Cognitive Impairment (MCI) or AD along with evidence of central nervous system amyloidosis (CSF PTau/Abeta 42 ratio > 0.028), a Mini Mental Status Examination (MMSE >24; no upper limit) and global clinical dementia rating (CDR) score between 0.5–1. Participants were excluded if MRI imaging was contraindicated, or were unable to tolerate MRI, or had a history of seizure disorder. Participants were on stable medications for cognitive impairment for a month prior to TMS treatment.

Twenty-nine participants were referred from the clinic with 7 excluded due to no confirmed diagnosis (*n* = 5) or implanted device (*n* = 2). Twenty-two participants were contacted, 7 were excluded due to advanced dementia, and one could not be reached. Fifteen participants were screened, with 4 participants excluded due to low MMSE score, and one patient withdrew prior to baseline visit. Ten participants were enrolled in the study (see consort diagram, Supplementary Figure S1) and all 10 completed the study.

### Neuropsychological testing and depression screening

All participants underwent a neuropsychological battery that included different versions of RBANS (Randolph et al., 1998). Participants received either A (baseline) or B (follow-up) versions of RBANS. Cognitive outcome measures were specifically chosen to minimize learning effects. Total and index scores for all domains were calculated for each participant including Immediate Memory Index, Visuospatial/Constructional Index, Language Index, Attention Index, and Delayed Memory Index.

All participants completed GDS-15 (De Craen et al., 2003) at baseline and 6-weeks after treatment. Scores range from 0 to 15, and higher scores indicate a greater likelihood that the participant requires further assessment for depression.

### Image acquisition and processing

All participants’ imaging was performed on a 3T Siemens Skyra Scanner. During MRI scanning, participants were instructed to rest quietly and stay awake until the end of the examination. T1-weighted image scan parameters included: echo time (TE) = 2.5 ms, matrix = 256×256, 1 mm slice thickness. Resting state functional MRI (rs-fMRI) scan parameters: T2-star echo-planar imaging sequence over an 8-min with 3.5 × 3.5 × 3.5 mm voxels, TR = 2,800 ms, TE = 27 ms, flip angle = 90°, 128 volumes/run. Diffusion weighted imaging or Diffusion tensor imaging (DTI) parameters included: 2 mm x 2 mm x 2 mm voxels, FOV = 25.6 cm, matrix = 128 mm x 128 mm, slice thickness = 2.0 mm, with one non-zero *b*-value of *b* = 1,000, 40 directions, and gap = 0.0 mm. The de-identified images were uploaded into a cloud-based platform for detailed structural-functional connectivity analyses using the Omniscient Infinitome software (Sydney, Australia), which uses machine learning-based brain image processing (Yeung et al., 2021). MRI was reviewed by the study neurologist for any lesions or strokes that may impact safety of the participant prior to start of TMS treatment.

#### Functional anomaly detection

The Infinitome tool creates a machine learning-based, subject specific version of the Human Connectome Project-Multimodal Parcellation version 1.0 (HCP-MMP1) atlas based upon diffusion tractography structural connectivity (Yeung et al., 2021). An HCP-MMP1 atlas in Neuroimaging Informatics Technology Initiative Montreal Neurological Institute (NIFTI MNI) space is warped onto each brain and the structural connectivity calculated between every pair of this atlas and a set of ROI (Regions of Interest) containing eight subcortical structures per hemisphere and the brainstem based on the streamlines which terminated within an ROI. The personalized patient atlas is used to subset the resting-state and CSD (Constrained spherical deconvolution-based) tractography data to create structural and functional connectivity matrices. Analytics are performed on both DTI and rs-fMRI. When these matrices are compared to other individuals using machine-learning, an output of structural and functional anomaly matrices demonstrates the abnormal connectivity in this subject’s brain. Outlier detection using a tangent space connectivity matrix is performed by comparing results with a subset of 200 normal HCP subject fMRI samples to determine the range of normal correlations for each region of interest in an LSBN. Abnormal connectivity is determined as a 3-sigma outlier for that correlation, after excluding the highest variance 1/3 of pairs, to further reduce the false discovery rate (Allan et al., 2019; Briggs et al., 2018a; Kuiper et al., 2020; Ren et al., 2020; Sheets et al., 2020).

### Personalized target selection

#### Pre-specified target

Preliminary research within our center using Infinitome in AD (*n* = 4; NCT04563767; Rosenbloom et al., 2021) and dementia with Lewy Body (DLB, *n* = 6; NCT04773041; Kashyap et al., 2022) identified right temporal area G dorsal (TGd) within the default mode network had a high functional anomaly burden. We selected this target for treatment with TMS *a priori* so that all participants regardless of their functional anomaly burden at this parcellation site received treatment at this target.

#### Personalized targets based on functional anomaly burden

Two additional targets were selected for each participant based upon high functional anomaly burden, as defined as >3 anomalies for each parcellation within the LSBNs (Young et al., 2023). These were ranked based upon the number of anomalies in a descending order. Selection of targets were based on three criteria: (1) highest number of anomalies, (2) accessible to TMS (superficial), and (3) clinically safe (e.g., considerations for effects on hearing). Each participant’s targets were selected after a discussion with the investigators and study physician prior to treatment. Targets were identified using .csv files from the Infintiome platform export and uploaded as NIFTI volumetric objects, co-registered to their T1 file, into the Localite Navigator (Bonn) image guidance platform.

### Study intervention

All iTBS sessions were completed with MagVenture TMS Therapy® (MagPror30/Theta Burst option) using a B65-coil-FDA cleared coil. Participants were administered 5 sessions of neuroimaging-guided (Localite Neuronavigator) accelerated iTBS per day for a total of 5 days in 2 weeks (see details in Figure 1). Three targets were stimulated at each session with a minimum of 2 min between targets and 45 min to an hour between sessions. All targets were stimulated with an 80% resting motor threshold, obtained at start of first day of treatment, using a visual observable motor response in the hand. iTBS was performed at 3-pulse 50 Hz bursts with 40 trains with an inter-train interval of 8 s for a total of 1,200 pulses, with a total of 18,000 pulses a day and 90,000 pulses for the treatment. The protocol was modified and adapted from SAINT protocol (Cole et al., 2020) and in consultation with collaborators (Young et al., 2023). The study aimed to mirror the SAINT protocol in the number of pulses per day and total pulses (90,000). Localite Neuronavigation (Bonn) tool was utilized for precision targeting through trackers on patient’s heads and coil with real-time feedback on location.

### Hypotheses and statistical analysis

The primary aim of the study was to estimate the effect of fc-rs-fMRI -guided TBS on connectivity dysfunction within the temporal area G dorsal (TGd) in persons with early-stage AD. We counted the number of parcellations whose connectivity with TGd was anomalous (≥3 standard deviations beyond a normal connectivity value, using the conservative connectome) and statistically compared this number pre- and post-intervention within person.

Additionally, the study had three exploratory aims: to estimate the effect of fc-rs-fMRI -guided TBS in persons with early-stage AD on (1) RBANS scores, (2) GDS score, and (3) two additional dysfunctional parcellations within DMN, CEN, and SN. RBANS total score and indices were calculated, and item-level differences were examined but not tested for statistical difference. The four most frequent parcellations (RTGd, L8Av, L55b, and L8BL) were chosen for the third exploratory aim. All statistical comparisons were made on pre -post-intervention data within person using the Wilcoxon signed-rank test, which is a nonparametric alternative to the paired t-test; given the small sample size (*n* ≤ 10), the assumptions of the paired t-test could not be verified. Multiple sensitivity analyses were performed and are described below.

## Results

### Subject characteristics

Demographics and baseline characteristics are presented in Table 1. The mean age of 10 participants who completed the study was 67.6 years, and 70% were male. All participants were non-Hispanic/Latino and of white race. The mean MMSE score was 26.3, and all participants had a rating of 0.5 CDR at baseline. Regarding concomitant medications, 7 participants were on donepezil, 1 on rivastigmine for cognitive impairment or dementia while two were not taking any dementia medication. Two participants were on an anti-depressant, while two participants were taking escitalopram for anxiety.

**Table 1:** Demographics and study population.

	Mean (SD) or *N* (%)
**Age**	67.6 (8.5)
**Sex**	
Male	7 (70%)
Female	3 (30%)
**Race**	
White	10 (100%)
**Ethnicity**	
Not Hispanic/Latino	10 (100%)
**Education**	
Bachelors/Associates	6 (60%)
Masters/PhD	4 (40%)
**Cognition**	
MMSE	26.3 (1.2)
Global CDR score	0.5 (0)

SD, standard deviation; N, number of participants; MMSE, mini mental status examination; CDR, clinical dementia rating.

### Unique targets identified using functional imaging

Supplementary Figure S1 provides a list of targets uniquely identified and personalized for treatment that had a high functional anomaly burden. Most common targets selected were left area 8A ventral [L8Av, (*n* = 7)], Left area 55b [L55b, (*n* = 4)], Left area 8B lateral [L8BL, (*n* = 3)] and right area superior frontal language [RSFL, (*n* = 2)]. Three participants had the same combination of targets for treatment (RTGd, L8Av and L55b). RTGd was pre-specified for all participants regardless of their anomaly burden. Other unique targets selected were left area 46 (L46), right area inferior 6–8 (Ri6-8), right superior 6–8 (Rs6-8) and right area 10 dorsal (R10d). Several parcellations were anomalous, but not selected due to a deeper location or clinical concerns regarding hearing [e.g., Left parietal area G superior (PGs), Left temporal area 1 anterior (TE1a)].

### Pre-post changes in functional anomaly burden

There were no significant differences between pre- and post-treatment (primary outcome) with respect to anomaly burden in targeted areas (see Table 2). Mean number of anomalous connections with RTGd was 25 for both pre- and post-treatment in all participants (*n* = 10) regardless of completed treatment at RTGd site. When limited to participants who completed treatment at this site, results were non-significant [mean (SD), pre – 21 (13), post – 24 (17), *p* = 0.99] as well. At an individual level, 43% (*n* = 3) participants showed a difference of ≥10 decreased anomalous connections, while 29% (*n* = 2) participants showed a difference of >27 increased anomalous connections after treatment with RTGd. Exploratory analysis looking at other commonly targeted areas L8Av, L55b and L8BL showed no significant differences in number of anomalous connections between pre- and post-treatment. With L8Av treated participants (*n* = 7), 57% (*n* = 4) of participants showed a difference of >13 increased anomalous connections after treatment.

**Table 2:** Pre-post treatment change in number of abnormal connections.

Area	Pre	Post	Difference	*N*	*p*-value*
RTGd	25 (14)	25 (15)	0.5 (25)	10	0.99
L8Av	38 (21)	40 (14)	2.3 (29)	10	0.8
L55b	16 (21)	17 (14)	0.3 (21)	10	0.99
L8BL	22 (18)	20 (9.3)	−1.3 (21)	10	0.99

*p-values from Wilcoxon signed-rank test.

N, number of participants; RTGd, right temporal area G dorsal; L8Av, left area 8A ventral; L55b, left area 55b; L8BL, left area 8B lateral.

### Pre-post changes in functional outcomes in early AD

Differences in RBANS indices are presented in Table 3. Exploratory analysis showed improvement in one of the indices, the mean attention index score (pre/post diff = 9.7, *p =* 0.01). Eighty percent of the participants showed an improved attention index score after treatment (Supplementary Figure S2). Story memory, part of the immediate memory index, showed higher scores in 70% of participants, though the specific index was not significant. Overall, we saw no difference in RBANS total score, or the other 5 indices (Table 3).

**Table 3:** Pre-post treatment changes in functional outcomes in early AD.

Test	Pre	Post	Difference	*P*-value*
RBANS total	81.4 (11.8)	84.3 (11.1)	2.9 (7.7)	0.43
RBANS immediate memory index	74 (18)	77 (18)	2.2 (14)	0.57
RBANS visuospatial/constructional index	86 (20)	88 (16)	2.8 (17)	0.84
RBANS language index	88 (9)	90 (5.6)	2 (7.7)	0.41
RBANS attention index	88 (21)	98 (21)	9.7 (8)	**0.01**
RBANS delayed memory index	70 (23)	68 (22)	−2.2 (19)	0.51
Geriatric Depression Scale	1.3 (1.3)	1.5 (1.7)	0.2 (1.9)	0.83

*p-values from Wilcoxon signed-rank test.

RBANS, repeatable battery for the assessment of neuropsychological status.

Participants were also evaluated for depression using GDS. GDS scores were normal at baseline, and no change was noted in the mean GDS scores after treatment (Table 3).

### Adverse events

No serious adverse events were reported. The most common treatment-related adverse event was facial or jaw twitching when participants (*n* = 10) were receiving TMS treatment at RTGd target. Participants experienced facial or jaw twitching with each treatment at RTGd target. No residual effects or pain were noted. Two participants were unable to tolerate the full stimulation intensity, and discontinued treatment at RTGd only, and one participant was anxious with the coil on their face and did not complete the treatment at this target. A few other mild adverse events were noted [headache (*n* = 1), tearing of the eye (*n* = 1), facial twitching at other sites (*n* = 2), scalp pain (*n* = 1)]. One participant had a spell of altered consciousness, between treatment days (not on a TMS treatment day) and treatment was paused. Participant completed the treatment protocol following evaluation for seizure and cardiac conditions with negative findings.

## Discussion

We performed a pilot study of fc-rs-fMRI -guided TMS to treat specific parcellations within dysfunctional LSBNs in 10 early-stage biomarker-confirmed AD subjects. Our clinical trial methodology was unique in that we utilized an fc-rs-fMRI -guided, personalized theta burst TMS treatment in a neurodegenerative disease process. This therapeutic approach has been successfully used to treat patients with refractory depression (Cole et al., 2020) but has not been adequately explored in persons with dementia. Furthermore, we identified novel HCP-defined parcellations uniquely impacted by AD that have not been previously described in the literature. The precuneus, a cortical hub within the DMN, is most consistently associated with dysfunctional DMN connectivity in AD as demonstrated both by brain FDG-PET (Strom et al., 2022) and fc-rs-fMRI imaging (Yokoi et al., 2018) and has served as a target in other TMS treatments studies in AD (Koch et al., 2022). Our study has revealed AD is a disease that extends beyond the precuneus, involving area right TGd, left 8Av and left 55b within the middle frontal gyrus, area 8BL, and right SFL. Specifically, area 8Av is considered to have effective connectivity with memory related posterior cingulate cortex regions (Leech and Sharp, 2014), a key node within DMN (Buckner et al., 2008) along with connections with anterior cingulate and parietal cortex. An understanding of AD at the level of the HCP parcellation may provide valuable insights into the clinical phenotype and future treatment of this condition (Ren et al., 2020).

This investigation of 10 early-stage AD participants failed to meet the primary outcome measure of demonstrating significant changes in LSBNs between baseline and 6 weeks. However, there were clinically significant improvements compared to baseline on the RBANS attention index score. TMS did not result in any improvements in other areas of cognition or in the total RBANs score, unlike the recent rTMS study which showing that targeted hippocampal-cortex connectivity improved memory domain for ADAS-Cog (Jung et al., 2024) or with iTBS in terms of delayed recall (Wu et al., 2020) in actively treated patients with AD. Due to experimental limitations, we did not have a comparison sham placebo group, which does not rule out the possibility that there may have been efficacy relative to a placebo (e.g., sham coil). For instance, findings of LSBN stability in the treatment group could be a favorable finding when compared to a placebo group in which LSBN functional connectivity is worsening.

Potential explanations for the improvement on the attention index score without any effect on LSBNs may be due to the stimulation of our targeted regions resulting in remote stimulation of other areas or LSBNS (e.g., dorsal and ventral attention networks) that were not included in our analysis (Cole et al., 2020). Certain treatment targets such as area 8Av with its effective connections with posterior cingulate cortex may have had an impact on improving attention (Hahn et al., 2007). In addition, we did not assess immediate post- TMS treatment effects on cognition or on fMRI in this study, which likely limited our ability to capture immediate effects of TMS treatments (Wu et al., 2020). Future studies are needed to better understand whether maintenance dosing is necessary to maintain favorable cognitive benefits in early-stage AD (Koch et al., 2022; Wu et al., 2024). It is crucial to account for learning effects associated with cognitive testing in any longitudinal study. In our study, to minimize this practice or learning effects we utilized different versions of the RBANS.

In the 10 early AD subjects evaluated, there were no significant adverse events associated with TMS, supporting this treatment as relatively low risk compared to the AMAs. Overall, our findings are consistent with what has been described in the TMS literature (Blumberger et al., 2018; Cole et al., 2020). The most common side effect involved jaw twitching when the TMS was targeted to area TGd, an adverse effect that limited our ability to fully dose certain subjects. Because of this frequent unpleasant effect, we will avoid this region in future treatment trials.

Strengths of our study included the enrollment of AD-biomarker confirmed subjects from a community neurology clinic. In addition, we piloted an innovative non-invasive neurostimulation treatment approach combining fc-rs-fMRI targeted TMS treatment to dysfunctional LSBNs in a progressive neurodegenerative disorder. To our knowledge, this is one of the first studies to utilize multimodal imaging and functional connectivity for personalized TMS in AD. This enhances the precision and personalization of TMS treatment offering greater accuracy than traditional scalp measurement targeting. Additionally, the study leveraged the Human Connectome Project (HCP), which provides high granularity in defining regions of interest (Glasser et al., 2016). For instance, the dorsolateral prefrontal cortex (DLPFC), a common TMS target, includes 13 distinct functional regions linked to multiple large-scale brain networks (Rosen et al., 2021). Furthermore, the imaging protocol can be easily added to a clinical MRI session, taking only an additional 15 min (Rosenbloom et al., 2021).

Study limitations relate to the pilot nature of this clinical trial and the small number of subjects with high education who also lack diversity, the absence of a sham-control group, and a relatively short treatment interval of 6 weeks, which may not have been adequate to capture immediate or delayed treatment responses. As mentioned previously, the jaw twitching related to the stimulation of area RTGd may have led to certain subjects receiving a subtherapeutic dose of this treatment. The TMS intervention in this study involved 2 research assistants per subject and took greater than 5 h to administer, which may impact translation to clinical practice. Finally, the precision-medicine approach to choosing parcellations based on associated functional connectivity anomalies resulted in an inconsistency of target regions treated between experimental subjects and reduced our power to detect differences for these regions. Moreover, there may have been instances where stimulation of one region may have activated an LSBN whereas stimulation of a second area may have led to an inhibitory response in that given network. For future trials, we hope to recruit a larger number of subjects from multiple sites, evaluate both an immediate and delayed neuropsychological outcome, and stimulate a single area of interest rather than targeting three different areas at a given time.

## Conclusion

Fc-rs-fMRI guided TMS may have potential therapeutic benefits on cognitive performance in early-stage AD through the modulation of LSBNs. Relative to other AD therapeutics, this treatment was safe and well-tolerated in all participating subjects. Future randomized, sham-controlled clinical trials are necessary to effectively investigate the role of non-invasive neurostimulation as a potential adjunctive therapy in early-stage AD.

## Data Availability

The datasets presented in this article are not readily available because of ethical reasons. Requests to access the datasets should be directed to Bhavani Kashyap, bhavani.x.kashyap@healthpartners.com.
