# Supplementary material for: Open label pilot of personalized, neuroimaging-guided theta burst stimulation in early-stage Alzheimer’s disease
Source: Front Neurosci. 2024 Dec 9;18:1492428. doi: 10.3389/fnins.2024.1492428 (PMC11663868; doi:10.3389/fnins.2024.1492428)

Supplement

Figure S1: Study Consort Diagram. 10 enrolled participants received intermittent theta burst (iTBS) TMS treatment**.** Abbreviations: AD- Alzheimer's Disease, MMSE – Mini Mental Status Examination, TMS – Transcranial Magnetic Stimulation.

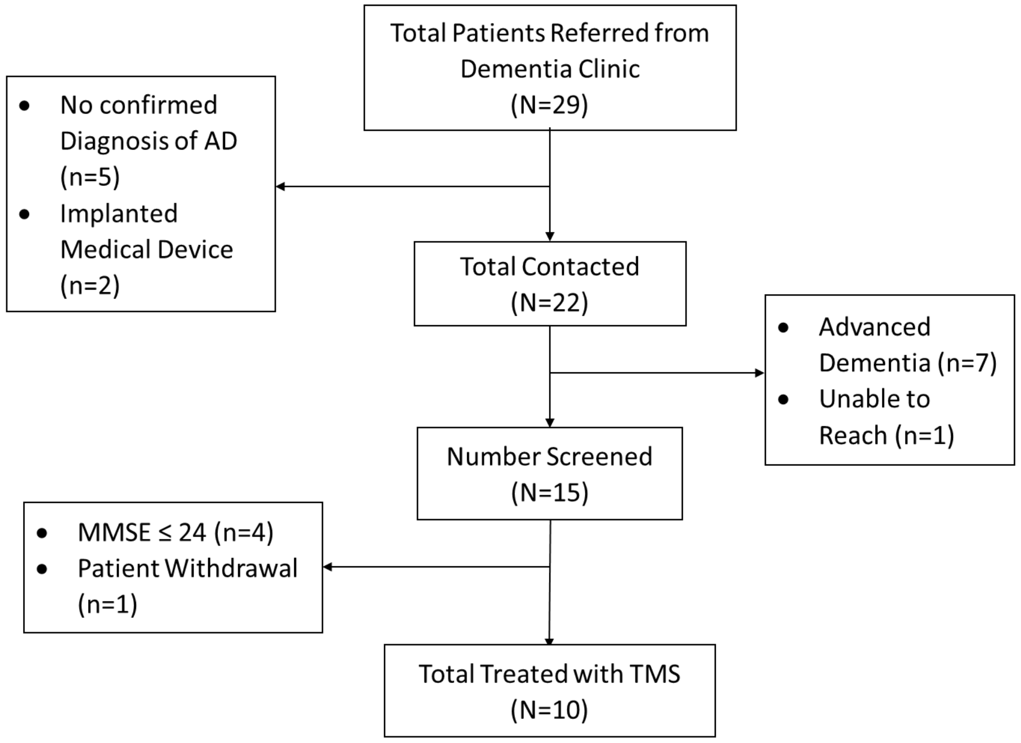

Table S1: Distribution of Combination of Targets for TMS treatment

Parcellations	N
L55b | L8Av | RTGd	3
L46 | L8Av | RTGd	1
L55b | L8BL | RTGd	1
L8Av | L8BL | RTGd	1
L8Av | RSFL | RTGd	1
L8Av | Ri6-8 | RTGd	1
L8BL | Rs6-8 | RTGd	1
R10d | RSFL | RTGd	1
N-number of Participants, L55b- Left Area 55b, L8Av – Left Area 8A ventral, RTGd – Right temporal area G dorsal, L46- Left Area 46, L8BL – Left area 8B lateral, RSFL – Right superior frontal language, Ri6-8- right area inferior 6-8, Rs6-8 - right superior 6-8, R10d - right area 10 dorsal	

Figure S2: TMS Impact on two Functional Measures in Early-Stage AD. 80% of the participants showed improved attention index and 70% of the participants showed improved scores for story memory after treatment. + RBANS Attention Index includes scores for Digit Span and Coding. ** RBANS Immediate Memory Index includes Story Memory. Abbreviations – TMS – Transcranial magnetic stimulation, AD - Alzheimer's disease, RBANS - Repeatable Battery for the Assessment of Neuropsychological Status

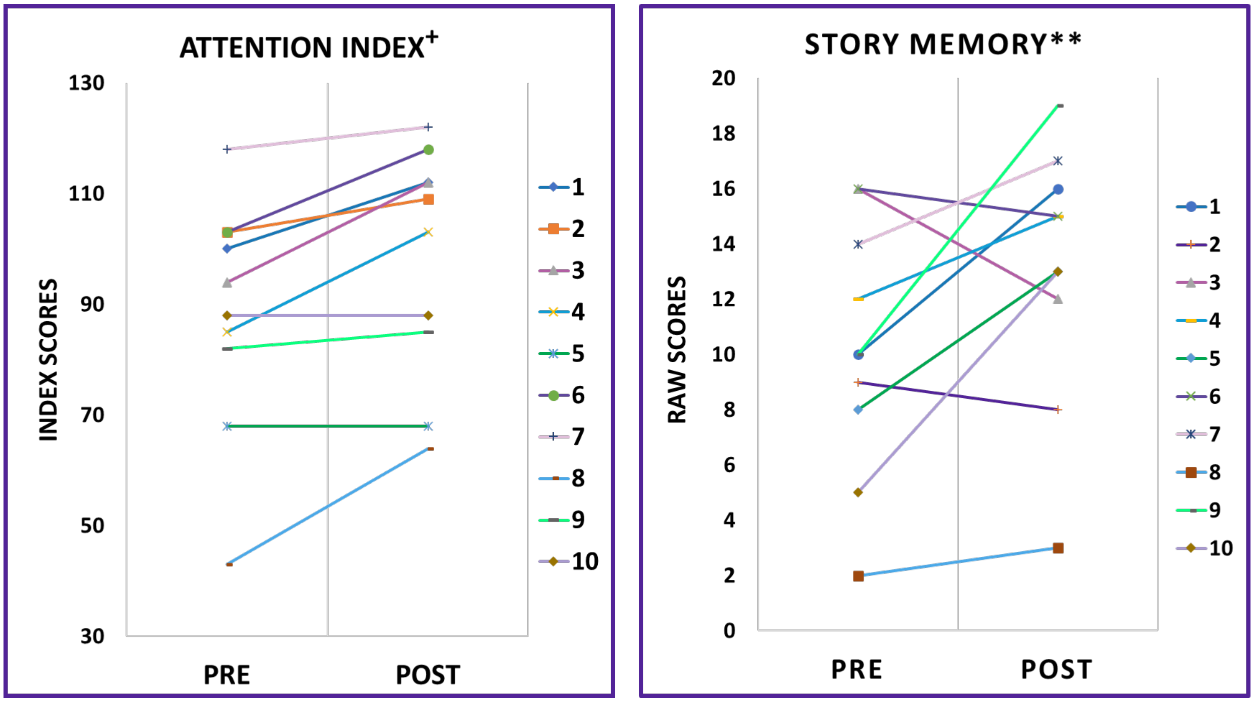

Supplement: Supplementary file 1 [file Data_Sheet_1.PDF]
